# Let us not neglect the impact of organizational culture on increasing diversity within medical schools

**DOI:** 10.1007/s40037-017-0342-4

**Published:** 2017-02-28

**Authors:** Kirsty Alexander, Jennifer Cleland, Sandra Nicholson

**Affiliations:** 1 Institute of Education in Medical and Dental Sciences, University of Aberdeen, Aberdeen, UK; 2 Centre for Medical Education, Barts and The London School of Medicine and Dentistry, London, UK

In this issue, Young et al. present a six-point framework for pipeline and program development, intended to increase diversity in Canadian medical schools [1]. Using a framework of knowledge translation, the authors emphasize the key role of collecting and monitoring longitudinal diversity-related data in designing, implementing and evaluating diversity-related initiatives. Young et al. argue the use of such data will not only improve best practice, but will also help build more tailored structures for lasting change.

We wholeheartedly agree that such data are essential. However, Young et al.’s focus on processes, new initiatives and structural change positions the article at the level of organizational and systems-level change. This is potentially problematic as, while systems-level changes may be an important initial step towards reducing functional barriers (to, in this case, increasing diversity within medicine), unless these barriers are also addressed at a cultural level, it is unlikely change will be effective, lasting or genuine [2, 3].

Institutional culture is loosely understood to be the shared assumptions, meanings, beliefs, understandings and ideas held within an organization, school or team – with a focus on values and judgements, rather than procedures and practices [4]. In established institutions, culture often errs towards stability and the status quo, largely allowing people to stay within their comfort zones and use established approaches rather than challenging these with innovation and growth [5]. Furthermore, medicine, and selection into medical school, are influenced by a pervasive culture based on meritocracy, where the prior academic achievements of applicants may be over-emphasized. Therefore, although an admissions system may be perceived to be effective by those in the institution, (for example, because it processes large numbers of applications efficiently or admits students who historically have low dropout rates), it may not be conducive to achieving ‘newer’ goals, such as those of increased diversity.

For example, and drawing on data from medical schools represented in the Young et al. study, Razack and colleagues [6–8] identified a potential conflict between medical schools’ definition of excellence and a culture that would permit goals for increased diversity (referred to here as widening access) to be met. As a group, medical schools’ discourses reflected that they valued ‘excellence in scholarship’ above other, potentially broader and more inclusive, notions of excellence [8]. Similarly, Alexander et al.’s [9] recent discourse analysis of UK medical school websites identified that, while the discourse of widening access for social mobility through academic meritocracy was dominant, a counter discourse for improving the workforce through increased diversity was marginalized. In neither study was widening access displayed as a strength, implying that while institutions may, at one level, acknowledge the need to widen access, and claim to put systems in place which support this, these systems may not be accompanied by a cultural shift towards truly embracing the value of diversity in medicine. Admissions practices, policies and their institutional interpretations may instead act as ‘filters’ [10] to intercept, moderate and even halt the effective implementation of widening access.

While acknowledgement of the need for cultural change may be especially important at a leadership level, as leaders exercise influence over their organization’s culture [11], at the same time, ‘top-down’ change is more likely to be sustained if it is embraced locally [2, 3]. This is clear in medical admissions. For example, Cleland et al.’s study [12] revealed that the aims of key stakeholders – the government policy makers and those who had to enact directives, the admissions deans – were often not in alignment with regard to widening access goals. Indeed, their data hinted that the political goal of widening access and medical education’s goal of producing the best doctors may conflict. Moreover, these and other data [13] indicate that those medical schools that embrace widening access at an organizational culture level may be more attractive to applicants from demographic groups currently underrepresented in medicine.

The aims and values of Young et al.’s proposed framework [1] need to align with the aims and values of those who would be working with it, or the framework’s impact is likely to be diluted or superficially incorporated for accountability purposes [12, 14–16]. However, only in their very last case study do Young and colleagues really stress the importance of developing markers and data collection processes in partnership with stakeholders. Leaving this as a later step implies to us an assumption that the stakeholders will share their views about increasing diversity. This may not be the case (see earlier) and it is much more likely that any framework will be implemented effectively if the views of stakeholders are sought very early on, to inform the processes of development, implementation and evaluation of change [17, 18].

Our final point is that, although cultural change in regard to medical admissions and increasing diversity may be notoriously difficult to enact, it is far from impossible. For example, in many countries medicine has become much more inclusive with regards to gender and some minority ethnic groups – but continues to lag behind in terms of increasing diversity on the grounds of socio-economic class [19–22]. It seems clear from patterns of success in increasing diversity that medical admissions are linked to wider societal issues. This is acknowledged by Young et al., who refer to the participation of Indigenous communities within medical education in the Canadian context. These wider issues must be taken into account when considering widening access and associated initiatives in any setting. However, it is also important to acknowledge very local contextual considerations. Each medical school has its own historical, social and local issues shaping the institutional culture and issues surrounding diversity and inclusion. Medical school A may embrace widening access while medical school B, in the same city, may be less overtly engaged, and the reasons for these differences are probably associated with medical school culture.

We call for more research exploring the influence of the particular cultural contexts: those of the wider socio-cultural, institutional and historical settings, and the complexities of the universities and medical schools within which medical admissions are situated and enacted. These issues may be effectively considered by a meso-level approach to promote and evaluate the necessary cultural change involved in establishing successful widening access programmes and policies [10, 12, 23].

In conclusion, coherent, evidence-informed frameworks with robust longitudinal data which allow us to evaluate progress are important to assessing the impact of widening access systems-level changes. However, unless there is an accompanying change in culture, we may be implementing superficial systems changes over a cultural status quo that is not conducive to achieving the goals of widening access. Only through a better understanding of the cultures within medical institutions that hamper increased diversity, can we target our efforts to implement lasting change to institutional systems and practices.
